# Platelet-Rich plasma as an effective alternative approach for improving endometrial receptivity - a clinical retrospective study

**DOI:** 10.5935/1518-0557.20200026

**Published:** 2020

**Authors:** Nilo Frantz, Marcelo Ferreira, Marcos Iuri Kulmann, Gerta Frantz, Adriana Bos-Mikich, Ricardo Oliveira

**Affiliations:** 1 Nilo Frantz Medicina Reprodutiva, Porto Alegre, RS, Brazil; 2 Instituto Ciências Básicas da Saúde, Universidade Federal Do Rio Grande do Sul, Brazil; 3 RDO, Diagnósticos Médicos Ltda., São Paulo, SP, Brazil

**Keywords:** Platelet-rich plasma, endometrium, receptivity

## Abstract

**Objective:**

The aim of the present case series was to describe our experience with the use of PRP on patients with refractory thin endometria.

**Methods:**

This retrospective analysis included 24 IVF cycles in which patients presenting different infertility factors received intrauterine PRP infusion prior to embryo transfer. Outcomes of interest were: clinical and ongoing pregnancies, miscarriages, and births.

**Results:**

54% of the cycles in which PRP was employed resulted in ongoing gestation or birth; 12.5% of the cycles ended in miscarriages.

**Conclusion:**

Our data suggest that PRP improves intrauterine receptivity to embryo implantation, regardless of whether the endometrium reached the appropriate growth for embryo transfer.

## INTRODUCTION

Despite the progress achieved in assisted reproduction technologies (ART), promoting embryo implantation seems to be an insurmountable challenge. Difficulties arise mainly from the fact that the elements working in conjunction for embryo implantation on natural conception have not been entirely elucidated. In ART cycles, there is a general idea that endometria with thickness ≥ 7 mm on the day human chorionic gonadotropin (hCG) administration are receptive endometria (Check *et al*., 1993). However, what does a receptive endometrium mean in terms of the uterine microenvironment? Evidence shows that endometrial epithelium, decidualized stromal cells, and immune cells produce and secrete paracrine signaling molecules known as growth factors and cytokines involved in decidualization, blastocyst attachment, and invasion (Duc-Goiran *et al*., 1999; Dey *et al*., 2004). White blood cells and platelets produce and secrete cytokines and growth factors, some of which known for their associations with embryo implantation in humans (Bos-Mikich *et al*., 2019).

Platelets are small anucleate cell fragments released from megakaryocytes found in bone marrow. Platelet cytoplasmic granules contain numerous proteins, several growth factors, and cytokines. When platelets are “activated”, these molecules are secreted and act synergistically to promote wound healing. In view of their healing and regenerative property, there is growing interest in the use of platelet-rich plasma (PRP) therapy to improve ART outcomes. PRP has been used particularly as an agent to induce endometrial growth in patients whose endometrial lining does not reach the desired thickness (>7mm) for embryo transfer. Results have shown improved ART outcomes (ongoing pregnancies and live births) associated with increased endometrial thickness after PRP administration (Chang *et al*., 2015; Tandulwadkar *et al*., 2017; Zadehmodarres *et al*., 2017; Molina *et al*., 2018).

This retrospective case series aims to report our experience with the use of PRP on patients whose endometria did not grow above 5mm after standard hormone treatment. Current reports in the literature showing encouraging pregnancy outcomes in this group of patients are discussed along with the factors potentially contributing to favorable outcomes.

## MATERIAL AND METHODS

This retrospective analysis included 24 treatment cycles performed on patients with and without a history of having undergone frozen embryo transfers (FET) at the Nilo Fratnz Medicina Reprodutiva Center between January and December 2018. Enrolled individuals gave written consent to joining the study. The study complied with the requirements set out in the STROBE Statement on Cohort Studies for a retrospective case series (Vandenbroucke *et al*., 2007).

**Inclusion criteria:** Patients with day-5 or day-6 good quality cryopreserved blastocysts graded ≥3BB (Gardner *et al*., 2000) with endometrium thickness below 5 mm during preparation for FET were included. Artificial endometrial preparation was performed with continuous oral estradiol valerate started on the day of the last menstrual cycle at an initial dose of 6mg. The first ultrasound scan was performed between seven and 10 days after the patients were started on estradiol valerate. When the endometrium did not grow to appropriate thickness, the estradiol valerate dose was increased to 8mg daily. Patients with thin endometria persisting after 14 to 17 days of estradiol valerate were given intrauterine PRP. A volume of 0.5 ml of final PRP preparation was administered into the uterine cavity of these patients with an embryo transfer catheter. PRP was administered every second day, for a total of three infusions. After the third PRP infusion, patients started using 800mg/day vaginal natural micronized progesterone. After the fifth day of progesterone administration the embryos were thawed and transferred.

**Outcomes of interest:** clinical and ongoing pregnancies, miscarriage, and birth.

**PRP preparation from whole blood:** PRP was prepared following a published protocol (Akhundov *et al*., 2012) with minor modifications. Patient whole blood was collected into 3.8% sodium citrate-containing Vacutainer tubes, transferred to centrifuge tubes, and centrifuged at 280g for 12 minutes at room temperature. PRP was harvested from the layer above the buffy coat and was transferred to a new centrifuge tube. After a second centrifugation (280g/10 minutes), PRP was collected and split into three samples. One was used immediately after preparation and the other two were kept at room temperature until use.

## RESULTS

Table 1 lists patient characteristics and the results of 24 poor prognosis IVF cycles (21 patients) due to refractory endometrium, the number of previous failed IVF cycles, and endometrial thickness status prior to PRP administration. A total of 16 clinical pregnancies (66.7%) were achieved in the present case series. Thirteen cycles (54%) resulted in ongoing pregnancies or live births, while three cycles ended in miscarriage.

**Table 1:** Patient characteristics, endometrial thickness prior to PRP administration and outcomes of 24 FET cycles

	OGP/Birth	Miscarriage	Non-pregnant
**No. of cycles (%)**	13 (54.2%)	3 (12.5%)	8 (33.3%)
**Mean age (range)**	34.9 (23-41)	34.3 (33-37)	33.7 (28-38)
Prior failed IVF attempts			
**1**	8	2	2
**2**	4	-	2
**3**	1	-	2
**4**	-	1	1
**5**	-	-	1
**Endometrial thickness (mm)**			
**3 – 4**	5	2	2
**4 – 5**	8	1	6

The majority of the cycles that resulted in ongoing pregnancy or birth occurred after a single failed previous cycle without PRP administration. Ten women had more than one infertility factor. Causes of infertility were numerous and varied, and involved male, female, or a combination of factors.

Three patients underwent two cycles with PRP administration. One female had one live birth after each cycle; the other two had miscarriages in one cycle and did not reach full pregnancies. The two miscarriages occurred when the women were six and seven weeks pregnant.

The three patients had miscarriages between week 6 and 7 of pregnancy received euploid embryos, an indication of good embryo quality.

### Embryo transfer and embryo characteristics

Embryo quality was high in all 24 cycles; only blastocysts graded AA, AB, or BA (Bl1) and BB or CB (Bl2) were transferred.

Table 2 shows that most of the patients achieving pregnancy after PRP administration were transferred a single embryo. Transferring one or two embryos resulted in similar pregnancy rates. There were no multiple pregnancies.

**Table 2:** Transfer and embryo characteristics.

	OGP/Birth	Miscarriage	Non- pregnant
**No. of cycles (%)**	13 (54.2%)	3 (12.5%)	8 (33.3%)
**No. Embryos transferred**			
**1**	8	3	6
**2**	5	0	2
**Mean**	1.38	1	1.25
**Blastocyst grade at transfer**			
**Bl1**	5	1	1
**Bl2**	3	2	5
**1Bl1 + 1Bl2**	1	-	2
**2Bl1**	2	-	-
**2Bl2**	2	-	-
**PGT-A**			
**Yes (%)**	6 (46.1%)	3 (100%)	6 (75%)
**No (%)**	7 (53.9%)	0 (0%)	2 (25%)

PGT-A was performed in the embryos of 15 cycles, six of which resulted in ongoing pregnancies and live births.

## DISCUSSION

The present report describes a case series of 24 IVF cycles in women given PRP due to poor endometrial growth after conventional estradiol/progesterone administration. Our results showed a remarkable effect of PRP in patients presenting different causes of infertility. More than 60% of the cycles resulted in clinical pregnancies, of which 54% moved on to ongoing pregnancies or live births. A common factor among all cycles was that the endometrium of the patients did not grow as expected after hormonal stimulation. Despite controversies in literature, thin endometria (<6mm) have been associated with decreased pregnancy and live birth rates (Dickey *et al*., 1993; Isaacs *et al*., 1996; Reuter *et al*., 1996; Warrington *et al*., 2008). A comprehensive review on different strategies to improve endometrial receptivity was published a few years ago (Garcia-Velasco *et al*., 2016). However, as the authors mentioned, there is no single efficient approach to treat all forms of refractory endometria. In addition, there is no strong evidence to support the use of one intervention over another.

However, after the first report by a Chinese group (Chang *et al*., 2015) on the use of PRP to promote endometrial growth in humans, others followed to demonstrate remarkable effects of this product on pregnancy and live birth rates on selected groups of patients (Bos-Mikich *et al*., 2018). A critical review of the reports cited above showed that the authors did not follow a standard protocol for PRP preparation or administration. In addition, the only trait their patients had in common was the fact that their endometria did not grow satisfactorily after conventional hormonal stimulation. These observations suggest that PRP used on a rather general population of infertile women promoted endometrial receptivity, rather than endometrial growth.

Several reports suggested that PRP components such as growth factors and cytokines act in conjunction to prepare the endometrium for pregnancy and favor implantation (von Wolff *et al*., 2000). Endometrial receptivity depends on several factors, among them the absence of pathogens that may interfere with embryo adhesion and implantation. Chronic endometritis is a highly prevalent condition among IVF patients. However, diagnosis is not always clear and endometritis is generally asymptomatic, which makes treatment ineffective. Anti-microbial anti-bacterial agents present in platelet granules may act on silent pathogens interfering with embryo implantation.

Expression of adhesion molecules by endometrial cells is another important factor in endometrial receptivity and embryo implantation. For example, certain integrins exhibit a regulated expression pattern throughout the menstrual cycle and may be reduced in infertile women (Lessey *et al*., 2000). The complex of factors contained in PRP may induce adhesion factor gene expression in endometrial cells, promoting endometrial receptivity, without necessarily promoting cell proliferation, as shown by Marini *et al*. (2016) in an *in vitro* model. However, the exact mode of action of PRP components on the endometrial lining remains unknown.

With that in mind, the aim of the present case series was not to control or measure endometrial growth, but to evaluate the effect of PRP administration in patients without an adequate endometrial lining for embryo transfer. In IVF treatments, a thin endometrium has been associated with lower pregnancy rates. Endometria deemed good for embryo transfers should present a triple-line pattern and thickness >7-8mm in ultrasound examination (Richter *et al*., 2007; Zhao *et al*., 2012; Kasius et al., 2011). The association between having a thin endometrium and achieving low pregnancy rates was also observed in a recent study of natural cycle IVF cycles and fresh embryo transfers (von Wolff et al., 2000). Having an appropriate endometrial lining is undoubtedly tied to presenting better chances of achieving pregnancy. However, endometrial thickness is not the only element at play, particularly when the findings described in hormone-stimulated IUI treatments are considered. After extensive literature reviews and analyses, authors found no significant correlation between endometrial thickness and pregnancy rates (Weiss *et al*., 2017) during IUI. It was suggested that embryos generated after IUI were more robust *in vivo* than the ones derived from IVF treatments and less susceptible to high oxygen exposure that occurs in thin endometria (Weiss *et al*., 2017).

We did not include a control group in our study, in which patients presenting poor endometrial growth underwent embryo transfers without PRP administration. As a norm, embryos are cryopreserved whenever endometrial growth does not reach a desired thickness and patients start a new hormonal treatment for endometrial preparation. Thus, this is not an analytical cohort study, where cases are described in comparison to a control group. Considering that we do not have a group to compare against patients given PRP, we cannot draw strong conclusions on how PRP administration affected endometrial receptivity, considering the different etiologies of infertility. However, our data showed that the majority of the patients who received PRP achieved pregnancy and 54% of the cycles in which PRP was employed resulted in ongoing pregnancies or live births.

Another important point to take into account in successful pregnancy is embryo quality. It is clear that more ongoing pregnancies or live births were achieved after the transfer of a single high-grade embryo (AA, AB or BA) when compared with transfers featuring a “second best” (BB or CB) embryo. However, it is worth pointing out that embryo quality measured based on euploidy status did not seem to have a significant effect on pregnancy rates. The transfer of euploid embryos did not result in higher pregnancy rates than the transfer of untested blastocysts. In addition, the three patients that had miscarriages between week 6 and 7 of pregnancy received euploid embryos, an indication of good embryo quality.

On the other hand, the fact that miscarriages occurred after 6 and 7 weeks of pregnancy may suggest that the embryos did not survive during or soon after the establishment of the placenta. By the beginning of the second month of gestation, the chorionic cavity should be covered by a great number of secondary and tertiary villi attached to the maternal decidua. The capillary system that develops in the core of the villi must soon come in contact with the capillaries of the connecting stalk and embryo, giving rise to a primitive embryo-maternal vascular system. This primitive exchange system is paramount for embryo survival through the later stages of development, and mishaps at this time may lead to embryo death before fetal life. To corroborate the idea that miscarriage may be a failure on the implantation process independent from the effects of embryo quality or endometrial thickness, von Wolff *et al.* (2000) did not find a relationship between miscarriage rates and endometrial thickness in a robust study on women undergoing natural cycle IVF.

In conclusion, the present report aimed to call attention to the positive effects of PRP intrauterine infusion in patients with refractory endometria. Our results suggested that PRP acts mostly by triggering endometrial receptivity, rather than promoting endometrial growth. Further randomized controlled studies are necessary to confirm this hypothesis.
